# Increased sympathetic modulation in breast cancer survivors determined by measurement of heart rate variability

**DOI:** 10.1038/s41598-022-18865-7

**Published:** 2022-08-29

**Authors:** Karolina Majerova, Milan Zvarik, Itay Ricon-Becker, Tsipi Hanalis-Miller, Iveta Mikolaskova, Vladimir Bella, Boris Mravec, Luba Hunakova

**Affiliations:** 1 Institute of Immunology, Faculty of Medicine, Comenius University in Bratislava, Bratislava, Slovakia; 2 Cancer Research Institute, Biomedical Research Center, Slovak Academy of Sciences, Bratislava, Slovakia; 3 Department of Nuclear Physics and Biophysics, Faculty of Mathematics, Physics and Computer Science, Comenius University, Bratislava, Slovakia; 4 School of Psychological Sciences, Tel-Aviv University, Tel-Aviv, Israel; 5 Department of Clinical Genetics and Department of Mammology, St. Elisabeth Cancer Institute, Bratislava, Slovakia; 6 Institute of Physiology, Faculty of Medicine, Comenius University in Bratislava, Bratislava, Slovakia

**Keywords:** Medical research, Oncology

## Abstract

Experimental and clinical studies have shown that the sympathetic nervous system (SNS) stimulates cancer progression and reduces the efficacy of oncological treatment. These effects may be reduced by pharmacological and psychotherapeutical approaches attenuating SNS tone. Therefore, it is necessary to identify those cancer survivors whose sympathetic modulation is excessively increased. For determination of SNS modulation, non-invasive method of heart rate variability (HRV) is widely used. In our study, HRV was determined from 5-min heartbeat recordings in healthy volunteers and in women with benign or malignant breast neoplasias, both in newly diagnosed patients and in women after initial treatment. We showed impaired cardio-vagal regulation in breast cancer patients (linear methods) and also found the increased sympathetic modulation indicated by the non-linear (the symbolic dynamics 0V%) parameter. This non-linear HRV analysis seems to be more sensitive than the linear one, indicating significant differences also in survivors after initial therapy in comparison to healthy controls. The lower sample entropy revealed reduced complexity in heart rate control in both breast cancer survivors groups. These findings suggest that HRV detection represents an inexpensive, easy, and reliable method for identification of those patients with breast cancer whose sympathetic modulation is significantly increased and in which the interventions, aimed at normalizing the balance in the autonomic nervous system (e.g. psychotherapy, biofeedback, treatment by β-blockers) may be the most effective.

## Introduction

Stress is now accepted as a factor that potentiates the progression of cancer^1^. This effect is mediated mainly by activation of the sympathetic nervous system (SNS)^2^. The role of SNS in cancer is a domain of the new scientific discipline, the so-called neurobiology of cancer^3^. Data from studies related to neurobiology of cancer have elucidated the mechanisms and pathways that mediate the stimulating effects of the SNS on cancer. It has been shown that mediators of the SNS, epinephrine and norepinephrine, potentiate cancer growth. In addition, these mediators also reduce the effectiveness of conventional cancer treatment^4^.

Clinical studies and metanalysis have shown that cancer-stimulating effect of stress is especially significant in subgroup of cancer patients with the highest “stress” score that corelates with the extent of SNS tone^1^. Therefore, we suggest that stress-reducing approaches might be most efficient in cancer patients with highest SNS modulation. Determination of sympathetic cardiac modulation might be therefore used for identification of cancer patients in which also psychological and pharmacological approaches reducing SNS modulation might be useful besides standard oncotherapy.

There are several methods reducing the adverse effects of the increased SNS tone on the tumor micro- and macro-environment in cancer patients. Psychotherapy has been used for decades in clinical practice, and in some cases was suggested to improve cancer patient’s survival^5^. In addition, several retrospective studies have shown that propranolol, reducing the effect of the SNS mediators on effector cells, may slow cancer progression^6^. The first prospective studies investigating the effect of propranolol on the course of cancer have yielded encouraging results^7,8^. However, not all studies that have examined the effect of psychotherapy or propranolol on the course of cancer have provided consistent results^6^. This is due to several factors. One potential explanation for such inconsistencies is the fact that the extent of the SNS activity in cancer patients can vary significantly^9^. Therefore, it is necessary to identify those cancer patients whose SNS activity is significantly increased. In these patients, the procedures aimed at reducing the SNS activity (e.g. psychotherapy, biofeedback, β-blocker treatment) might be the most effective, may considerably reduce the stimulatory effect of the SNS on cancer progression, and possibly improve the effectiveness of conventional cancer treatment^1^.

The extent of the SNS activity in humans can be determined by several methods. For instance, it can be determined by measuring the levels of epinephrine and norepinephrine in plasma or by measuring the electrical activity of sympathetic nerves. However, these procedures are methodologically complicated, time consuming, and expensive. Therefore, to meet the needs of clinical practice, it is necessary to choose a method that is simple, non-invasive, time-saving, and inexpensive. This is met by a simple and non-invasive method determining the heart rate variability (HRV), using electrocardiogram (ECG) or photoplethysmography (PPG) recording.

HRV analysis is based on the determination of successive heartbeat intervals variability^10^, which is believed to reflect the balance between the activities of the sympathetic (SNS) and parasympathetic (PNS) nervous systems, relating to the activation and regulation of stress^11^. HRV can be extracted from ECG using basic analytical methods, such as time domain, frequency domain, nonlinear, and dynamic systems methods^12^. The obtained data can be used to assess the SNS modulation, reflecting the spontaneous variations of sympathetic neural activities about its mean^13^. Recently, several published studies have shown altered HRV in breast cancer patients. However, these studies were mostly focused on the role of the vagus nerve in relation to breast cancer^14^ or altered autonomic control of the cardiovascular system^15^. Nevertheless, altered HRV might also reflect increased stress and other factors that are associated with diagnosis and cancer treatment^16^.

Therefore, in our study, we used HRV to determine both the parasympathetic and sympathetic modulation in healthy volunteers, in patients with benign breast tumors, and in patients with breast cancer (survivors). Patients with malignant tumors were divided into two groups, a group of newly diagnosed breast cancer (active disease- M-A), and patients who had already completed initial treatment for breast cancer (M-CIT).

## Patients and Methods

### Sample characteristics

The study was approved by the St. Elisabeth Cancer Institute (Slovakia) and Sheba Medical Center (Israel) Ethics Review Board for human study, and informed consent was obtained from all participants of the study.

The study sample consisted of 69 women (54 from Slovakia, and 15 from Israel, for characteristics of the patients see Table 1). Four groups were identified based on the presence of malignancy. The control group (C) of healthy women consisted of 21 randomly selected women aged 62.71 (± 12.6) years with no malignancy, determined by clinical examination, mammography and ultrasonography. They did not have previous cancer history. The group of patients with benign tumor (B) consisted of 13 women aged 56.92 (± 11.4) years. The group of survivors with active breast cancer (M-A) comprised 20 women with the newly diagnosed breast cancer aged 58.85 (± 11.57) years, and the group of survivors after completed initial treatment (M-CIT) consisted of 15 women minimally one year after primary anticancer therapy aged 68.13 (± 12.0) years. There were no differences in age, BMI and respiratory rate between these groups (Table 1).

**Table 1:** Proband’s characteristics (mean ± SD).

Variables	Control (C) (N = 21)	Benign (B) (N = 13)	Survivors (M-A) (N = 20)	Survivors (M-CIT) (N = 15)	p
Age (years)	62.71 ± 12.61	56.92 ± 11.43	58.85 ± 11.57	68.13 ± 12.01	0.062
Height (m)	1.64 ± 0.05	1.67 ± 0.08	1.62 ± 0.07	1.61 ± 0.03	0.064
Weight (kg)	68.76 ± 14.87	73.54 ± 14.52	68.05 ± 12.67	64.53 ± 7.67	0.338
BMI (kg m^−2^)	25.44 ± 5.03	26.5 ± 4.92	26.04 ± 4.45	24.86 ± 2.97	0.77
RR (Hz)	0.23 ± 0.05	0.26 ± 0.06	0.25 ± 0.06	0.26 ± 0.06	0.152

*M-A* survivors with active cancer, *M-CIT* survivors after completed initial treatment, *BMI* body mass index, *RR* respiration rate.

Participants with hypertension, cardiovascular disease, diabetes mellitus (type I and II) and acute or chronic infection were excluded from the investigation, as well as recordings with ectopic beats > 5% of all beats.

### Ethical approval

All procedures performed in studies involving human participants were in accordance with the ethical standards of the institutional and/or national research committee and with the 1964 Helsinki declaration and its later amendments or comparable ethical standards.

### Heart rate variability recordings

Heart rate variability was assessed in a sitting position in a 23 ± 2 °C room temperature. After a 5-min period at rest, the measurements with duration of 5 min were performed by iFeel HRV Sensor (iFeel Healthy Inc., Israel) and emWave Pro (HeartMath, LLC, Boulder, Colorado), with automatic pulse wave detection and calibration. Plethysmograph derived data have been shown to highly and significantly correlate with ECG-derived heart rate measurement during the rest periods^17^. The stored data were further checked for the presence of ectopic beats and artefacts, and the artefact-free 5-min sequences were analyzed by Kubios 2.2 (Kuopio, Finland)^18^ system to achieve 51 HRV parameters for further evaluation. The non-linear analysis including pattern classification was processed by a symbolic dynamics algorithm according to Porta et al. (2001)^19^. The summary of all executing HRV parameters is in the Table 2.

**Table 2:** Summary of executing HRV parameters according to^18,19^.

Parameter	Units	Description
**Time domain**		
Mean RR	ms	The mean of RR intervals
SDNN	ms	Standard deviation of RR intervals
Mean HR	min^−1^	The mean heart rate
STD HR	min^−1^	Standard deviation of instantaneous heart rate values
Min HR	min^−1^	Minimum HR computed using N beat moving average (N = 5)
Max HR	min^−1^	Maximum HR computed using N beat moving average (N = 5)
RMSSD	ms	Square root of the mean squared differences between successive RR intervals
NN50	Beats	Number of successive RR interval pairs that differ more than 50 ms
pNN50	%	NNxx divided by the total number of RR intervals
HRV triangular index		The integral of the RR interval histogram divided by the height of the histogram
TINN	ms	Baseline width of the RR interval histogram
**Frequency-domain—spectrum Welch’s periodogram**		
Peak frequency	Hz	VLF, LF, and HF band peak frequencies
Absolute power	ms^2^	Absolute powers of VLF, LF, and HF bands
Absolute power	log	Natural logarithm transformed values of absolute powers of VLF, LF, and HF bands
Relative power	%	Relative powers of VLF, LF, and HF bands
Normalized power	n.u	Powers of LF and HF bands in normalized units (VLF not included)
Total power	ms^2^	Total absolute powers (VLF + LF + HF)
LF HF ratio		Ratio between LF and HF band powers
**Nonlinear**		
SD1	ms	In Poincaré plot, the standard deviation perpendicular to the line-of-identity
SD2	ms	In Poincaré plot, the standard deviation along the line-of-identity
SD2 / SD1		Ratio between SD2 and SD1
ApEn		Approximate entropy
SampEn		Sample entropy
DFA, α1		In detrended fluctuation analysis, short term fluctuation slope
DFA, α2		In detrended fluctuation analysis, long term fluctuation slope
D2		Correlation dimension
Lmean	Beats	Mean line length
Lmax	Beats	Maximum line length
REC	%	Recurrence rate
DET	%	Determinism
ShanEn		Shannon entropy
**Symbolic dynamics**		
0V		Number of patterns with zero variation
1V		Number of patterns with one variation
2LV		Number of patterns with two like variations
2UV		Number of patterns with two unlike variations
0V (%)	%	Relative number of patterns with zero variation
1V (%)	%	Relative number of patterns with one variation
2LV (%)	%	Relative number of patterns with two like variations
2UV (%)	%	Relative number of patterns with two unlike variations
**Indices**		
PNS index		Parasympathetic nervous system activity compared to normal resting values
SNS index		Sympathetic nervous system activity compared to normal resting values
Stress index		Square root of Baevsky’s stress index

#### Linear analysis—time-domain analysis

Time-domain analysis worked with the calculation of beat-to-beat differences in the heartbeat duration and consisted of the standard deviation of the interval between heartbeats (SDNN) and the root mean square of successive differences (RMSSD). The parameter RMSSD reflects the parasympathetic heart-rate regulation^12^.

#### Linear analysis—frequency-domain analysis

Spectral analysis of HRV quantified an amplitude of oscillations in the distinct frequency bands: VLF: 0–0.04 Hz, LF: 0.04–0.15 Hz, and HF: 0.15–0.4 Hz^12^. Prior to analysis, the slow fluctuations were filtered using smoothness priors detrending^20^. The time series of the beat-to-beat heart rate recordings were converted by cubic spline interpolation with the 2 Hz sampling frequency. The power spectrum was assessed using Welch’s periodogram based on Fast Fourier Transformation with 256 sample segment-length and 50% overlap. The high-frequency band of the HRV reflecting respiratory sinus arrhythmia was evaluated as an index of cardiac vagal regulation, while the LF band was considered to reflect the predominant sympathetic regulation.

#### Non-linear analysis—symbolic dynamics

We determined the symbolic dynamics of HRV introduced by Porta et al. (2007)^21^. It was based on the transformation of the time series into series of symbols with various range levels of RR-interval durations. Subsequently, the triplets of heartbeats were classified into different patterns according to variations as follows: 0 V% (zero variation), 1 V% (one variation), 2LV% (two like variations) and 2UV% (two unlike variations). The occurrence of these patterns was subsequently evaluated^21,22^. It is important to note that the 0 V% (1 V%) and 2UV% (2LV%) parameters can reflect the cardiac-linked sympathetic and parasympathetic regulation, respectively^22–24^.

#### Nonlinear analysis—entropy

Sample entropy (SampEn) measured the complexity and irregularity of the biological signals. It is independent of the length of the analyzed time series and reduces the calculation bias^25^. Healthy regulatory mechanisms are characterized by the higher entropy values and indicate a greater irregularity and complexity in the regulation of biological functions^19^.

### Statistical analysis

Descriptive statistical data were expressed as the mean (SD) or median (Q1, Q3). The Shapiro–Wilk test was used to test the normality of HRV indices. Dependent variable analyses for the HRV parameters were separately conducted using the parametric and nonparametric tests. One-way ANOVA was used to calculate normal data and the Fisher’s least significant difference (LSD) was used for the multiple comparisons between groups. The Kruskal–Wallis test was used to analyze the non-normal data. Finally, the separate multiple logistic regression models were used with survivors with active cancer (M-A) or after treatment (M-CIT) as an outcome and with each significant HRV parameter set as a predictor while adjusted for age, body mass index (BMI), mean heart rate (HR), and respiration rate (RR). In both cases, the group of healthy women and the group of patients with benign tumor, represented the negative outcomes. In the regression analysis the group of survivors with the newly diagnosed breast cancer, as well as those after treatment were set were set as a positive outcome. The data were analyzed using the R environment for statistical computing^26^, and a value of p < 0.05 was considered statistically significant.

## Results

In our work we examined the time- and frequency- domain HRV indices as well as the nonlinear parameters as sample entropy and the symbolic dynamics parameters 0V (%), 1V (%), 2LV (%) and 2UV (%). The choice of selected parameters was established by their known association with sympathetic and parasympathetic cardiac modulations.

### Linear analysis

**Figure 1:**
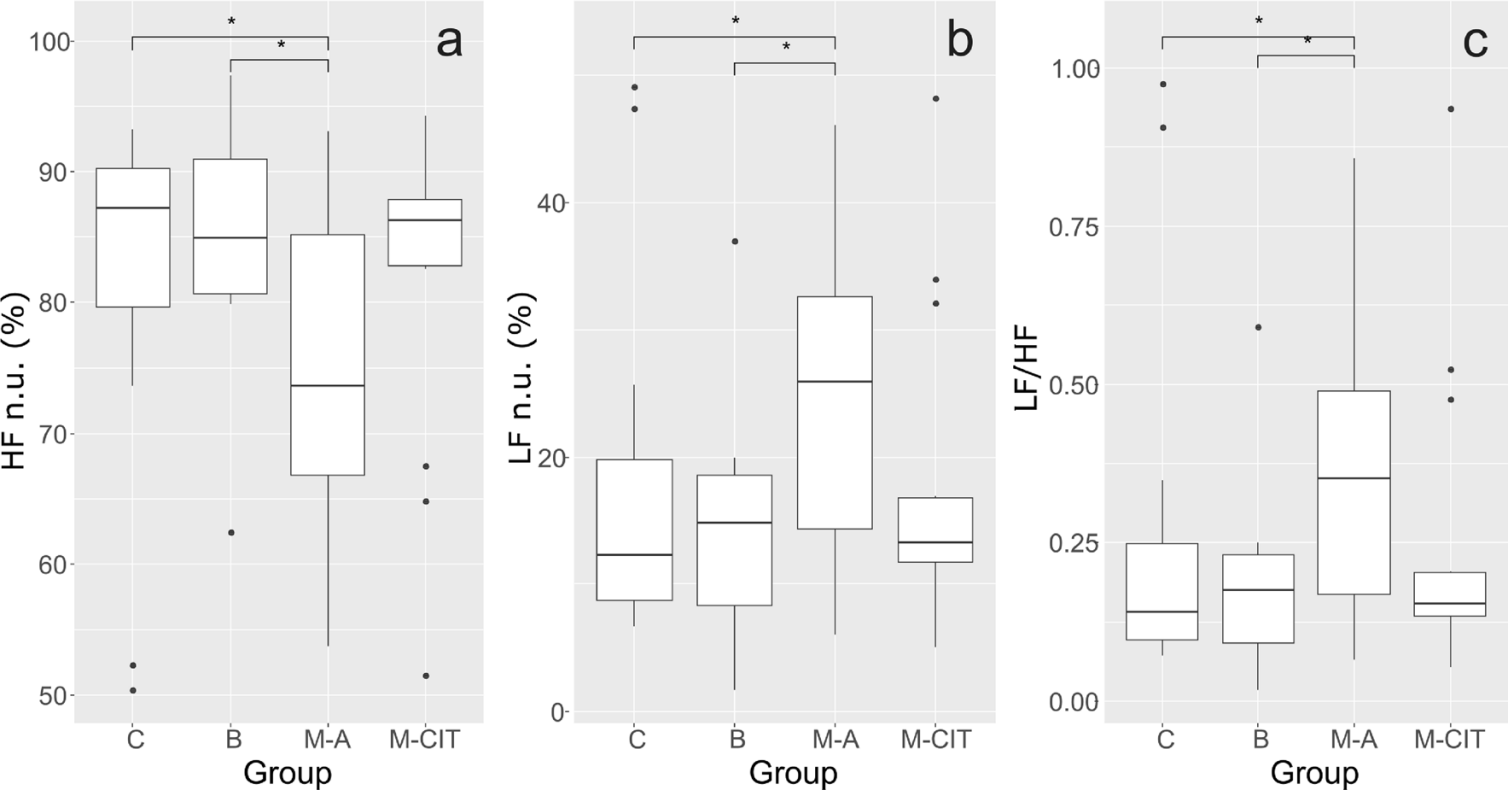
Intergroup comparison of the HF n.u. (**a**), LF n.u. (**b**) and LF/HF ratio (**c**). Brackets indicate significant differences between groups (*p < 0.05; Kruskal–Wallis test). *C *control group, *B *benign tumor, *M-A* survivors with active cancer, *M-CIT* survivors after completed initial treatment.

There were no significant differences in the time-domain analysis between studied groups (Table 3). However, the frequency domain analysis of HRV revealed the lower parasympathetic activity of active breast cancer patients, indexed by the HF normalized power, when compared to healthy women (p = 0.011) and patients with benign tumors (p = 0.027; Fig. 1A, Table 3). The normalized power of the LF region was increased in the active breast cancer group in comparison to healthy women (p = 0.011) and patients with a benign tumor (p = 0.026; Fig. 1B, Table 3). Similarly, the normalized LF/HF ratio, often used to quantify the degree of sympatho-vagal balance, showed the same trend as the LF n.u. region (p = 0.011 resp. p = 0.027; Fig. 1C, Table 3).

**Table. 3:** Comparison of HRV selected parameters among studied groups.

Variables	Control (C) (N = 21)	Benign (B) (N = 13)	Survivors (M-A) (N = 20)	Survivors (M-CIT) (N = 15)	p
0V (%)	16.17 ± 6.44	17.18 ± 9.08	24.11 ± 9.75	26.23 ± 9.7	**0.002**
1V (%)	44.63 ± 3.58	43.51 ± 5.75	44.87 ± 3.85	43.9 ± 4.67	0.799
2LV (%)	9.66 (7.62, 13.8)	9.38 (7.07, 19.87)	7.33 (5.14, 8.4)	5.64 (3.75, 8.76)	**0.022**
2UV (%)	29.1 (24.44, 32.73)	26 (21.93, 29.01)	21.98 (17.94, 27.75)	23.26 (19.51, 29.24)	**0.036**
SDNN (ms)	19.08 (14.84, 31.74)	22.83 (13.86, 29.12)	18.04 (14.25, 21.13)	20.72 (13.56, 26.12)	0.703
Mean HR (bpm)	68.22 ± 8.42	68.4 ± 7.47	75.71 ± 9.74	67.69 ± 12.95	**0.043**
RMSSD (ms)	27.53 (22.77, 43.36)	32.03 (20.65, 41.1)	24.74 (21.27, 29.84)	31.21 (20.3, 38.39)	0.47
VLF (ms^2^)	0.63 (0.31, 1.16)	0.64 (0.34, 1.07)	0.88 (0.36, 1.65)	0.78 (0.41, 0.87)	0.897
LF (ms^2^)	57.83 (17.61, 110.97)	44.62 (13.95, 98.31)	54.53 (24.84, 102.36)	43.39 (21.7, 70.92)	0.828
HF (ms^2^)	201.74 (129.7, 585.7)	338.41 (109.5, 416.2)	155.57 (92.75, 238.6)	216.4 (115.0, 462.2)	0.393
HF n.u. (%)	87.62 (81.02, 90.24)	84.98 (80.68, 91.01)	73.63 (66.79, 85.2)	86.33 (82.81, 87.86)	**0.047**
TP (ms^2^)	264.6 (147.6, 740.9)	347.51 (132.4, 518.6)	220.15 (108.2, 343.5)	291.27 (133.4, 523.4)	0.631
LF/HF	0.14 (0.09, 0.23)	0.17 (0.09, 0.23)	0.35 (0.17, 0.49)	0.15 (0.13, 0.2)	**0.047**
SampEn	1.86 ± 0.21	1.75 ± 0.25	1.62 ± 0.22	1.6 ± 0.24	**0.002**

Values are expressed as mean (SD) with normal distribution or median (Q1, Q3) without normal distribution. Bold values of p indicate statistical significance (value of p < 0.05).

*M-A* survivors wit active cancer; *M-CIT* survivors after completed initial treatment, *0V* zero variation, *1V* one variation, *2LV* two like variations, *2UV* two unlike variations, *ApEn* approximate entropy, *bpm* beats per minute, *HF* high-frequency power, *HR* heart rate, *LF* low-frequency power, *LF/HF* ratio of low-frequency power to high-frequency power, *RMSSD* root mean square of successive interval differences, *SampEn* sample entropy, *SDNN* standard deviation of all normal-to-normal intervals, *TP* total power, *VLF* very low frequency.

### Nonlinear analysis

**Figure 2:**
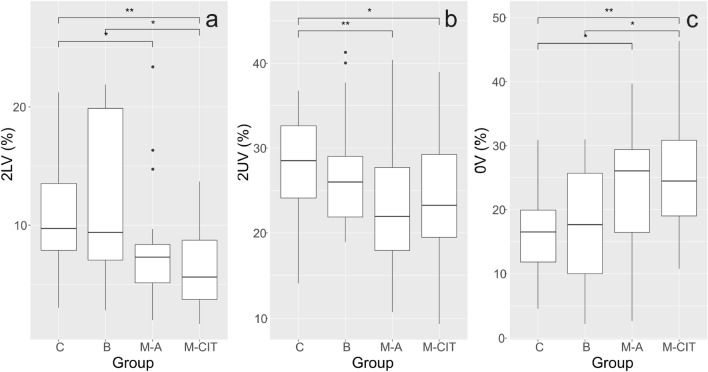
Intergroup comparison of the 2LV (%) (**a**), 2UV (%) (**b**) and 0V (%) (**c**) parameter. Brackets indicate significant differences between groups [*p < 0.05, **p < 0.01; Kruskal–Wallis test, LSD for (**c**)]. *C* control group, *B *benign tumor, *M-A* survivors with active cancer, *M-CIT* survivors after completed initial treatment.

Symbolic dynamics, namely 2LV (%) and 2UV (%) indices, revealed a decreased parasympathetic activity in the group of survivors with active breast cancer (p = 0.049; p = 0.009, resp.) and in the group of survivors after completed initial therapy (p = 0.007; p = 0.037, resp.) compared to the control group (Fig. 2A,B). On the other hand, the sympathetic activity in these groups measured by 0V (%), was increased in comparison to healthy women (p = 0.025; p = 0.006) (Fig. 2C). These characteristics also remained decreased (2LV%, 2UV%) or increased (0V%) in the survivor’s M-CIT group.

**Figure 3:**
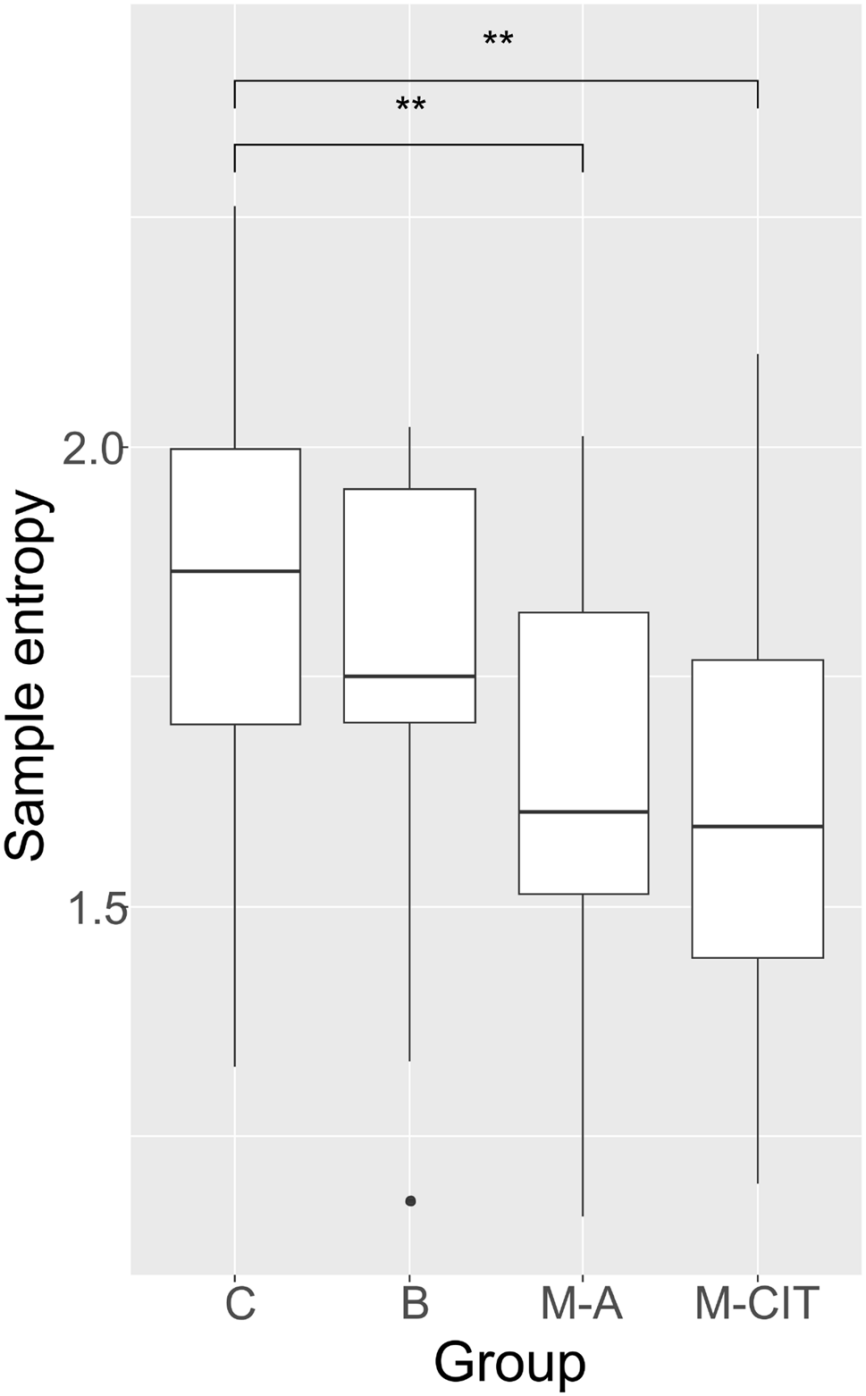
Intergroup comparison of the sample entropy. Brackets indicate significant differences between groups (**p < 0.01; LSD). *C *control group, *B *benign tumor, *M-A* survivors with active cancer, *M-CIT* survivors after completed initial treatment.

The complexity of autonomic nervous system activity was characterized by the sample entropy. This parameter was decreased in the group of survivors with active cancer (p = 0,007) and in the survivors after completed initial treatment (p = 0.007), when compared with the group of healthy women (Fig. 3).

Multiple logistic regression models were also conducted adjusting for age, BMI, mean HR, and mean RR (Table 4).

**Table 4:** Survivors regression models (adjusted for age, BMI, Mean HR, and RR).

Variables	Active cancer (M-A)				Completed initial treatment (M-CIT)			
	OR	95% CI for OR		p	OR	95% CI for OR		p
		Lower	Upper			Lower	Upper	
0V (%)	1.107	1.022	1.216	**0.019**	1.264	1.102	1.565	**0.007**
2LV (%)	0.886	0.752	1.017	0.110	0.705	0.519	0.884	**0.008**
2UV (%)	0.906	0.814	0.992	**0.046**	0.838	0.704	0.956	**0.021**
LF n.u. (%)	1.066	1.002	1.143	0.052	1.066	0.983	1.165	0.131
HF n.u. (%)	0.938	0.874	0.997	0.051	0.939	0.858	1.018	0.133
LF/HF	25.405	0.972	1229.272	0.068	25.121	0.396	2551.359	0.135
SampEn	0.032	0.001	0.497	**0.023**	0.009	0.000	0.288	**0.016**

Active cancer (M-A)—control and benign group as negative outcome, M-A group as positive outcome; completed initial treatment (M-CIT)—control and benign group as negative outcome, M-CIT group as positive outcome.

Bold values of p indicate statistical significance (value of p < 0.05).

*0V* zero variation, *2LV* two like variations, *2UV* two unlike variations, *CI* confidence interval, *HF n.u.* high-frequency normalized power, *HF/LF* ratio between LF and HF band powers, *LF* low-frequency normalized power, *OR* odds ratio, *SampEn* sample entropy.

The associations of active cancer with the relative number of patterns with zero variation (0V%), the relative number of patterns with two unlike variations (2UV%) and the sample entropy were significant in logistic regression analysis (Table 4). Interestingly, for each one-unit increase in 0V (%), the odds of having breast cancer increased by 10.7% (OR: 1.107, Table 4). For a one-unit increase in 2UV (%) and the sample entropy, the odds decreased by 9.4% (OR: 0.906) and by 96.8% (OR: 0.032), respectively.

If survivors after initial treatment group (M-CIT) was determined as a positive outcome in the models, the parameters 0V (%), 2UV (%), 2LV (%) and the sample entropy were significant, the effect being even more pronounced. Odds ratio (OR) for these parameters reached the values of 1.264, 0.705, 0.838 and 0.009, respectively (Table 4).

## Discussion

By using HRV measurement, we have shown altered cardio-vagal regulation in breast cancer survivors. Using the linear and non-linear HRV indices showed the increased sympathetic modulation in both malignant groups—newly diagnosed (M-A) and after completing initial therapy (M-CIT). Importantly, our findings indicate that the sympathetic modulation in breast cancer survivors might remain significantly increased. Therefore, approaches reducing prevalence of sympathetic cardiac modulation and stress might be useful in cancer survivors.

Our data has demonstrated that the measurement of HRV represents a feasible method for the identification of those breast cancer patients, who may benefit from approaches reducing the negative effects of stress on cancer. It is necessary to note that for appropriate determination of the extent of the PNS and SNS activity from HRV recordings, ECG must be measured for at least 5 min. In addition, it is necessary to use a device that enables simple manipulation with raw data from which HRV indices can be calculated.

In our study, evaluation of the time-domain (SDNN—Standard deviation of RR intervals and RMSSD—Square root of the mean squared differences between successive RR intervals) indices of HRV, reflecting the parasympathetic heart-rate regulation, did not show any significant changes between groups. These data correlate with findings of similar studies^14,15^, in which no significant differences were found between early-stage breast cancer patients and healthy controls. While these studies have shown no significant differences for the linear frequency domain indices, our spectral analysis identified the lower parasympathetic activity of breast cancer patients, indexed by HF n.u. region, and the increased sympathetic activity, indexed by LF n.u. region, as well as the LF/HF n.u. ratio. This can be attributed to different experimental setups, as their patients were measured in a supine position. However, the recent studies^27^ have also shown the increased sympathetic and decreased parasympathetic modulation, indexed by normalized LF n.u. and HF n.u. spectral parameters, as well as the sympatho-vagal balance (LF/HF n.u.) increased from ECOG1 to ECOG4 performance status, according to Eastern Cooperative Oncology (ECOG) Scale in breast cancer patients. Alterations in the autonomic nervous system activity are known contributors to cancer-related fatigue^28^. Anxiety and depression, so common in breast cancer patients, can affect their ANS and hemodynamic stability, which are important factors during surgery, reflecting different patterns of the ANS reactivity of anxious and depressed patients^29^.

Finally, insufficient heart rate adaptation to different challenges associated with breast cancer can be connected with altered cognitive-emotional regulatory circuitry^30^.

However, when we put these normalized spectral parameters (LF n.u, HF n.u. and LF/HF) into the logit regression model, the parameters were not correlated with the presence of malignancy. In the intergroup comparison, when considering mean values, there were significant differences only between survivors with active malignancy (M-A), but not in survivors after completing initial treatment (M-CIT) compared to healthy controls and/or patients with benign breast tumors. Based on our linear analysis it might seem that breast cancer survivors could retrieve their sympatho-vagal balance after the primary therapy. However, some literature has suggested that HRV is commonly lower post breast cancer surgery and follow-up treatment^31^. Palma et al. (2016)^32^ reported more pronounced changes in autonomic modulation with the longer postoperative period, including RMSSD, absolute power of HF, the standard deviation of Poincaré plot perpendicular (SD1) and along (SD2) to the line-of-identity and SDNN indices. In this study, probands were measured in a supine position for 30 min. As the time-domain analysis strongly depends on the length of HRV recordings, time-varying recordings cannot be compared. In addition, breast cancer survivors of that study had a significant proportion of comorbidities, which could affect the results. Breast cancer groups had hypertension (40 and 33.3%), hypercholesterolemia (6.7 and 13.3%), depression (6.7 and 20%) and diabetes (6.7 and 13.3%) in higher proportions than the control group. The higher resting HF was associated with longer overall survival in patients with recurrent or metastatic breast cancer^33^, and the lower HF power was previously observed in breast cancer survivors^31^, but normalized LF n.u. and HF n.u. were similar in breast cancer survivors and healthy age-matched controls. Using a linear analysis, we found the same in normalized power for breast cancer survivors after initial treatment (M-CIT), having the differences significant only for the group of patients with active breast cancer (M-A).

The linear methods can not properly characterize the complex dynamics of the autonomic nervous system activity, which is nonlinear in humans. Therefore, it is important to employ the novel nonlinear indices that could better reflect the complexity, irregularity and dynamic characteristics of the HRV signal. The intrinsic limitation of the LF power that mixes vagal and sympathetic controls led some researchers to utilize different approaches for the noninvasive evaluation of the sympathetic modulation^34,35^. From various possible approaches, where esp. QT interval variability is of interest^35^, we chose to use symbolic analysis series to quantify the prevalence of sympathetic or parasympathetic cardiac modulation in our study.

Although the spectral parameters did not show significant differences between survivors after initial therapy (M-CIT) and healthy controls/benign tumors, we assessed the non-linear parameters that identified the differences similar to the group of survivors with active breast cancer (M-A), suggesting that survivors after initial treatment (M-CIT) also maintain the impaired complex of cardio-vagal integrity. The symbolic dynamics reflecting the vagal/sympathetic regulation revealed the decreased parasympathetic (2LV%, 2UV%) and the increased sympathetic modulation (0V%) in the group of survivors with active disease and those with completed initial therapy in comparison to the control group. Similarly, the complexity of the ANS effects characterized by sample entropy was decreased in the group of patients with active malignancy (M-A) and in the patients after initial treatment (M-CIT), when compared to the group of healthy women. The analyses, according to Porta et al.^19^ was never performed before in cancer patients. Therefore, our preliminary pilot study^30^ and the present study can be considered original. Importantly, they are in agreement with others, mostly cardiovascular studies suggesting that the nonlinear HRV analysis is significantly superior to the linear one^36,37^.

In our study, the associations between the HRV parameters and breast cancer survivorship were tested using multiple logistic regression models. Since a logit analysis is adjusted for age, BMI, mean HR, and mean RR, it is considered more precise in the estimation of the association of the HRV parameters to observed outcomes. Such an approach has confirmed the superiority of nonlinear methods: while the linear parameters were not significant, the nonlinear parameters revealed the correlation between present or undergone malignancy and HRV integrity.

### Limitations of the study

Even if our study brings original data, there are several limitations that need to be mentioned. In the future studies, it will be useful to enlarge number of participants and to determine HRV during the course of breast cancer treatment to identify dynamics of changes of parasympathetic and sympathetic tone. In addition, there are many other interesting nonlinear indices (representative of fractality-multifractality, predictability, empirical mode decomposition, and Poincaré plot families—^38^ and representative of heart rate asymmetry—^39,40^) that could be assessed if having larger cohort.

## Conclusion

In conclusion, our data indicated that the non-linear evaluation of HRV, from 5-min ECG recordings might represent a simple and inexpensive method for the non-invasive assessment of the parasympathetic and sympathetic nervous system modulation in breast cancer survivors. Seemingly, it can be a more appropriate method for the evaluation of HRV than the linear analysis, as it showed the impaired complex cardio-vagal integrity also after initial anticancer therapy. Using this non-linear analysis might help to select breast cancer survivors that could most benefit from approaches aimed at reducing the SNS effects on tumor micro- and macroenvironment. Further research is thus warranted to assess the potential benefits of interventions for selected cancer patients according to their HRV. In such studies, standardized methodological factors such as body position, measurement length, and cognitive load during measurements, are imperative for producing interpretable and replicable results.

## Data Availability

The datasets generated during and/or analysed during the current study are available from the corresponding author on reasonable request.

## References

[1] Eckerling A, Ricon-Becker I, Sorski L, Sandbank E, Ben-Eliyahu S (2021). Stress and cancer: Mechanisms, significance and future directions. Nat. Rev. Cancer.

[2] Mravec B, Tibensky M, Horvathova L (2020). Stress and cancer. Part I: Mechanisms mediating the effect of stressors on cancer. J. Neuroimmunol..

[3] Mravec B (2021). Neurobiology of cancer: Definition, historical overview, and clinical implications. Cancer Med..

[4] Mravec B, Horvathova L, Hunakova L (2020). Neurobiology of cancer: The role of beta-adrenergic receptor signaling in various tumor environments. Int. J. Mol. Sci..

[5] Chen Y, Ahmad M (2018). Effectiveness of adjunct psychotherapy for cancer treatment: A review. Future Oncol..

[6] Mravec B, Tibensky M, Horvathova L (2020). Stress and cancer. Part II: Therapeutic implications for oncology. J. Neuroimmunol..

[7] De Giorgi V (2018). Propranolol for off-label treatment of patients with melanoma: Results from a cohort study. JAMA Oncol..

[8] Haldar R (2020). Perioperative COX2 and beta-adrenergic blockade improves biomarkers of tumor metastasis, immunity, and inflammation in colorectal cancer: A randomized controlled trial. Cancer.

[9] Wackerhage H (2022). Cancer catecholamine conundrum. Trends Cancer.

[10] Berntson GG (1997). Heart rate variability: Origins, methods, and interpretive caveats. Psychophysiology.

[11] Luo H (2019). Stress determined through heart rate variability predicts immune function. NeuroImmunoModulation.

[12] Heart rate variability: Standards of measurement, physiological interpretation and clinical use. Task Force of the European Society of Cardiology and the North American Society of Pacing and Electrophysiology. *Circulation* **93**, 1043–1065 (1996). 8598068

[13] La Rovere MT, Porta A, Schwartz PJ (2020). Autonomic control of the heart and its clinical impact. A personal perspective. Front. Physiol..

[14] Wu S, Chen M, Wang J, Shi B, Zhou Y (2021). Association of short-term heart rate variability with breast tumor stage. Front. Physiol..

[15] Arab C (2018). Cardiac autonomic modulation impairments in advanced breast cancer patients. Clin. Res. Cardiol..

[16] Grote S, Ricci JM, Dehom S, Modeste N, Sealy DA, Tarleton HP (2020). Heart rate variability and cardiovascular adaptations among cancer-survivors following a 26-week exercise intervention. Integr. Cancer Ther..

[17] Giardino ND, Lehrer PM, Edelberg R (2002). Comparison of finger plethysmograph to ECG in the measurement of heart rate variability. Psychophysiology.

[18] Tarvainen MP, Niskanen JP, Lipponen JA, Ranta-Aho PO, Karjalainen PA (2014). Kubios HRV—Heart rate variability analysis software. Comput. Methods Programs Biomed..

[19] Porta A (2001). Entropy, entropy rate, and pattern classification as tools to typify complexity in short heart period variability series. IEEE Trans. Biomed. Eng..

[20] Tarvainen MP, Ranta-Aho PO, Karjalainen PA (2002). An advanced detrending method with application to HRV analysis. IEEE Trans. Biomed. Eng..

[21] Porta A (2007). Complexity and nonlinearity in short-term heart period variability: Comparison of methods based on local nonlinear prediction. IEEE Trans. Biomed. Eng..

[22] Porta A (2006). Role of the autonomic nervous system in generating non-linear dynamics in short-term heart period variability. Biomed. Tech. (Berl).

[23] Visnovcova Z (2014). Complexity and time asymmetry of heart rate variability are altered in acute mental stress. Physiol. Meas..

[24] Tonhajzerova I (2016). Symbolic dynamics of heart rate variability - a promising tool to investigate cardiac sympathovagal control in attention deficit/hyperactivity disorder (ADHD)?. Can. J. Physiol. Pharmacol..

[25] Richman JS, Moorman JR (2000). Physiological time-series analysis using approximate entropy and sample entropy. Am. J. Physiol. Heart Circ. Physiol..

[26] R-project.org. R: *The R Project for Statistical Computing*. https://www.R-project.org/ (2021).

[27] Shukla RS, Aggarwal Y (2021). Fourier transform and autoregressive HRV features in prediction and classification of breast cancer. IETE J. Res..

[28] Crosswell AD, Lockwood KG, Ganz PA, Bower JE (2014). Low heart rate variability and cancer-related fatigue in breast cancer survivors. Psychoneuroendocrinology.

[29] Farbood A, Sahmeddini MA, Bayat S, Karami N (2020). The effect of preoperative depression and anxiety on heart rate variability in women with breast cancer. Breast Cancer.

[30] Hunakova L (2019). Cardiovagal regulation and transcutaneous pO2 in breast cancer patients—A pilot study. Neoplasma.

[31] Arab C (2016). Heart rate variability measure in breast cancer patients and survivors: A systematic review. Psychoneuroendocrinology.

[32] Palma MR (2016). The relationship between post-operative time and cardiac autonomic modulation in breast cancer survivors. Int. J. Cardiol..

[33] Giese-Davis J (2015). Higher vagal activity as related to survival in patients with advanced breast cancer: An analysis of autonomic dysregulation. Psychosom. Med..

[34] Guzzetti S (2005). Symbolic dynamics of heart rate variability: A probe to investigate cardiac autonomic modulation. Circulation.

[35] Porta A (2015). Autonomic control of heart rate and QT interval variability influences arrhythmic risk in long QT syndrome type 1. J. Am. Coll. Cardiol..

[36] Voss A, Schulz S, Schroeder R, Baumert M, Caminal P (2009). Methods derived from nonlinear dynamics for analysing heart rate variability. Philos. Trans. A Math. Phys. Eng. Sci..

[37] de Godoy, M. F. Nonlinear analysis of heart rate variability: A comprehensive review. *J. Cardiol. Ther.* **3**, 528–533. 10.17554/j.issn.2309-6861.2016.03.101-4 (2016).

[38] Maestri R (2007). Nonlinear indices of heart rate variability in chronic heart failure patients: Redundancy and comparative clinical value. J. Cardiovasc. Electrophysiol..

[39] Porta A, Guzzetti S, Montano N, Gnecchi-Ruscone T, Furlan R, Malliani A (2006). Time reversibility in short-term heart period variability. Comput. Cardiol..

[40] Guzik P, Piskorski J, Krauze T, Wykretowicz A, Wysocki H (2006). Heart rate asymmetry by Poincaré plots of RR intervals. Biomed. Tech. Biomed. Eng..

